# 1-[2-(4-Fluoro­benz­yloxy)-2-phenyl­ethyl]-1*H*-benzimidazole

**DOI:** 10.1107/S1600536808021326

**Published:** 2008-07-16

**Authors:** Özden Özel Güven, Taner Erdoğan, Simon J. Coles, Tuncer Hökelek

**Affiliations:** a Zonguldak Karaelmas University, Department of Chemistry, 67100 Zonguldak, Turkey; b Southampton University, Department of Chemistry, Southampton SO17 1BJ, England; c Hacettepe University, Department of Physics, 06800 Beytepe, Ankara, Turkey

## Abstract

The asymmetric unit of the title compound, C_22_H_19_FN_2_O, contains two independent mol­ecules. The planar benzimidazole ring systems are oriented with respect to the phen­yl/fluoro­benzene rings at dihedral angles of 31.10 (4)/45.17 (5) and 45.52 (5)/68.63 (5)°, respectively, for the two mol­ecules. In the crystal structure, inter­molecular C—H⋯N and inter­molecular C—H⋯N and C—H⋯F hydrogen bonds link the mol­ecules into a three-dimensional network. There are C—H⋯π contacts between the benzimidazole and fluoro­benzene rings and a π–π contact between the benzimidazole and phenyl ring systems [centroid–centroid distance = 4.575 (1) Å].

## Related literature

For general background, see: Brammer & Feczko (1988 ▶); Özel Güven *et al.* (2007*a* ▶,*b* ▶). For related literature, see: Song & Shin (1998 ▶); Freer *et al.* (1986 ▶); Peeters *et al.* (1979*a* ▶,*b* ▶, 1996 ▶); Caira *et al.* (2004 ▶).
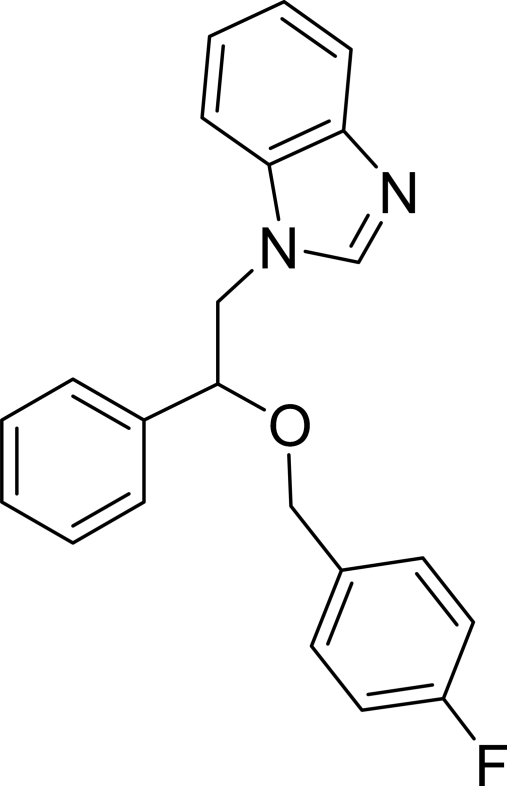


## Experimental

### 

#### Crystal data

C_22_H_19_FN_2_O
*M* *_r_* = 346.39
Monoclinic, 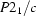

*a* = 12.6946 (2) Å
*b* = 18.1279 (4) Å
*c* = 15.3547 (3) Å
β = 95.747 (1)°
*V* = 3515.76 (12) Å^3^
*Z* = 8
Mo *K*α radiation
μ = 0.09 mm^−1^
*T* = 120 (2) K
0.55 × 0.15 × 0.14 mm

#### Data collection

Bruker–Nonius KappaCCD diffractometer
Absorption correction: multi-scan (*SADABS*; Sheldrick, 2007 ▶) *T*_min_ = 0.953, *T*_max_ = 0.988
41740 measured reflections
8050 independent reflections
5679 reflections with *I* > 2σ(*I*)
*R*_int_ = 0.065

#### Refinement

*R*[*F* ^2^ > 2σ(*F* ^2^)] = 0.055
*wR*(*F* ^2^) = 0.142
*S* = 1.07
8050 reflections
622 parameters
All H-atom parameters refined
Δρ_max_ = 0.43 e Å^−3^
Δρ_min_ = −0.43 e Å^−3^

### 

Data collection: *COLLECT* (Hooft, 1998 ▶); cell refinement: *DENZO* (Otwinowski & Minor, 1997 ▶) and *COLLECT*; data reduction: *DENZO* and *COLLECT*; program(s) used to solve structure: *SHELXS97* (Sheldrick, 2008 ▶); program(s) used to refine structure: *SHELXL97* (Sheldrick, 2008 ▶); molecular graphics: *ORTEP-3 for Windows* (Farrugia, 1997 ▶); software used to prepare material for publication: *WinGX* (Farrugia, 1999 ▶) and *PLATON* (Spek, 2003 ▶).

## Supplementary Material

Crystal structure: contains datablocks I, global. DOI: 10.1107/S1600536808021326/xu2436sup1.cif

Structure factors: contains datablocks I. DOI: 10.1107/S1600536808021326/xu2436Isup2.hkl

Additional supplementary materials:  crystallographic information; 3D view; checkCIF report

## Figures and Tables

**Table 1:** Hydrogen-bond geometry (Å, °) *Cg*2 is the centroid of the N1′/N2′/C1′/C2′/C7′ ring and *Cg*8 is the centroid of the C17′–C22′ ring.

*D*—H⋯*A*	*D*—H	H⋯*A*	*D*⋯*A*	*D*—H⋯*A*
C6—H6⋯N2′	0.97 (2)	2.62 (2)	3.498 (2)	150.5 (17)
C6′—H6*A*⋯N2^i^	0.964 (19)	2.459 (19)	3.345 (2)	152.7 (16)
C13—H13⋯F^i^	1.00 (2)	2.54 (2)	3.493 (2)	159.6 (16)
C19′—H19*A*⋯N2′^ii^	1.01 (2)	2.59 (2)	3.509 (2)	151.1 (16)
C5—H5⋯*Cg*8^iii^	0.95 (2)	2.662 (19)	3.514 (2)	149.0 (15)
C22′—H22*A*⋯*Cg*2^iv^	0.98 (2)	2.62 (9)	3.518 (2)	141.9 (16)

Symmetry codes: (i) 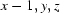; (ii) 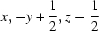; (iii) 
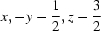
; (iv) 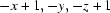.

**Fig. 1.**
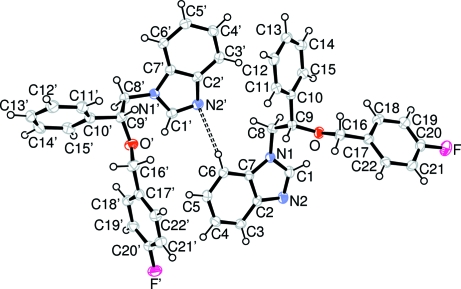
The molecular structure of the title molecule with the atom-numbering scheme. Displacement ellipsoids are drawn at the 50% probability level. Hydrogen bond is shown as double dashed line.

**Fig. 2.**
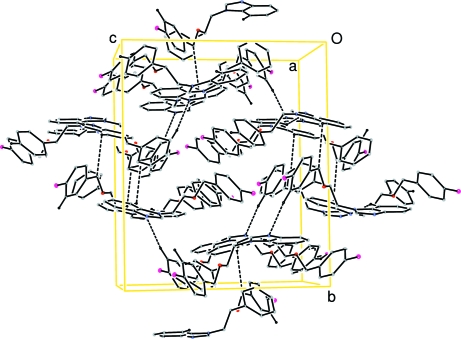
A packing diagram of (I). Hydrogen bonds are shown as dashed lines.

## supplementary crystallographic information

### Comment

In recent years, there has been increasing interest in synthesis of heterocyclic compounds having biological and commercial importances. Clotrimazole (Song & Shin, 1998), econazole (Freer *et al.*, 1986), ketoconazole (Peeters *et al.*, 1979*a*) and miconazole (Peeters *et al.*, 1979*b*) are well known imidazole ring containing, while itraconazole (Peeters *et al.*, 1996) and fluconazole (Caira *et al.*, 2004) are 1*H*-1,2,4-triazole ring containing, azole derivatives. They have been developed for clinical uses as antifungal agents (Brammer & Feczko, 1988). Lately, similar structures to miconazole and econazole have been reported to show antibacterial activity more than antifungal activity (Özel Güven *et al.*, 2007*a*,b). In these structures, benzimidazole ring has been found in place of the imidazole ring of miconazole and econazole. We report herein the crystal structure of title benzimidazole derivative.

The asymmetric unit of the title compound (Fig. 1) contains two independent molecules, in which the bond lengths and angles are generally within normal ranges. The planar benzimidazole ring systems are oriented with respect to the phenyl and fluorobenzene rings at dihedral angles of 31.10 (4)°, 45.17 (5)° and 45.52 (5)°, 68.63 (5)° for unprimed and primed molecules, respectively. Atoms C8, C9, C16 and C8', C9', C16' are -0.105 (2), 0.065 (2), -0.060 (2) and -0.163 (2), -0.088 (2), 0.009 (2) Å away from the ring planes of the corresponding benzimidazole, phenyl and fluorobenzene, respectively. So, they are nearly coplanar with the attached rings.

In the crystal structure, intramolecular C—H···N and intermolecular C—H···N and C—H···F hydrogen bonds (Table 1) link the molecules into a three dimensional network (Fig. 2), in which they may be effective in the stabilization of the structure. The C—H···π contacts (Table 1) between the benzimidazole and the fluorobenzene rings and a π-π contact between the benzimidazole and phenyl ring systems *Cg*1···*Cg*6^i^[symmetry code: (i) 1 - *x*, 1/2 + *y*, 1/2 - *z*, where *Cg*1 and *Cg*6 are centroids of the rings (N1/N2/C1/C2/C7) and (C10'-C15'), respectively] further stabilize the structure, with centroid-centroid distance of 4.575 (1) Å.

### Experimental

The title compound was synthesized by the reaction of 2-(1*H*-benzimidazol-1-yl) -1-phenylethanol (Özel Güven *et al.*, 2007*a*) with NaH and appropriate benzyl halide. A solution of alcohol (300 mg, 1.259 mmol) in DMF (2.4 ml) was added to NaH (63 mg, 1.574 mmol) in small fractions. The appropriate benzyl halide (238 mg, 1.259 mmol) in DMF (1.2 ml) was added dropwise. The mixture was stirred at room temperature for 2 h, and the excess hydride was decomposed with a small amount of methyl alcohol. After evaporation to dryness under reduced pressure, the crude residue was suspended with water and extracted with methylene chloride. The organic layer was dried over anhydrous sodium sulfate, and then evaporated to dryness. The crude residue was purified by chromatography on a silica-gel column using chloroform-methanol as eluent. Crystals suitable for X-ray analysis were obtained by the recrystallization of the ether from a mixture of hexane/ethyl acetate (1:2) (yield; 364 mg, 83%).

### Refinement

H atoms were located in difference syntheses and refined isotropically [C—H = 0.94 (2)–1.05 (2) Å; *U*_iso_(H) = 0.023 (5)–0.051 (6) Å^2^].

### Crystal data

**Table:**

C_22_H_19_FN_2_O	*F*_000_ = 1456
*M**_r_* = 346.39	*D*_x_ = 1.309 Mg m^−^^3^
Monoclinic, *P*2_1_/*c*	Mo *K*α radiation λ = 0.71073 Å
Hall symbol: -P 2ybc	Cell parameters from 8094 reflections
*a* = 12.6946 (2) Å	θ = 2.9–27.5º
*b* = 18.1279 (4) Å	µ = 0.09 mm^−^^1^
*c* = 15.3547 (3) Å	*T* = 120 (2) K
β = 95.747 (1)º	Rod-shaped, colorless
*V* = 3515.76 (12) Å^3^	0.55 × 0.15 × 0.14 mm
*Z* = 8	

### Data collection

**Table:**

Bruker–Nonius KappaCCD diffractometer	8050 independent reflections
Radiation source: fine-focus sealed tube	5679 reflections with *I* > 2σ(*I*)
Monochromator: graphite	*R*_int_ = 0.065
Detector resolution: 9.091 pixels mm^-1^	θ_max_ = 27.5º
*T* = 120(2) K	θ_min_ = 3.0º
φ and ω scans	*h* = −16→14
Absorption correction: multi-scan(SADABS; Sheldrick, 2007)	*k* = −23→23
*T*_min_ = 0.953, *T*_max_ = 0.988	*l* = −19→19
41740 measured reflections	

### Refinement

**Table:**

Refinement on *F*^2^	Hydrogen site location: inferred from neighbouring sites
Least-squares matrix: full	All H-atom parameters refined
*R*[*F*^2^ > 2σ(*F*^2^)] = 0.055	*w* = 1/[σ^2^(*F*_o_^2^) + (0.0671*P*)^2^ + 0.7368*P*] where *P* = (*F*_o_^2^ + 2*F*_c_^2^)/3
*wR*(*F*^2^) = 0.142	(Δ/σ)_max_ < 0.001
*S* = 1.07	Δρ_max_ = 0.43 e Å^−^^3^
8050 reflections	Δρ_min_ = −0.43 e Å^−^^3^
622 parameters	Extinction correction: SHELXL97 (Sheldrick, 2008), Fc^*^=kFc[1+0.001xFc^2^λ^3^/sin(2θ)]^-1/4^
Primary atom site location: structure-invariant direct methods	Extinction coefficient: 0.0237 (14)
Secondary atom site location: difference Fourier map	

### Special details

**Table:**

Geometry. All e.s.d.'s (except the e.s.d. in the dihedral angle between two l.s. planes) are estimated using the full covariance matrix. The cell e.s.d.'s are taken into account individually in the estimation of e.s.d.'s in distances, angles and torsion angles; correlations between e.s.d.'s in cell parameters are only used when they are defined by crystal symmetry. An approximate (isotropic) treatment of cell e.s.d.'s is used for estimating e.s.d.'s involving l.s. planes.
Refinement. Refinement of *F*^2^ against ALL reflections. The weighted *R*-factor *wR* and goodness of fit *S* are based on *F*^2^, conventional *R*-factors *R* are based on *F*, with *F* set to zero for negative *F*^2^. The threshold expression of *F*^2^ > σ(*F*^2^) is used only for calculating *R*-factors(gt) *etc*. and is not relevant to the choice of reflections for refinement. *R*-factors based on *F*^2^ are statistically about twice as large as those based on *F*, and *R*- factors based on ALL data will be even larger.

### Fractional atomic coordinates and isotropic or equivalent isotropic displacement parameters (Å^2^)

**Table:**

	*x*	*y*	*z*	*U*_iso_*/*U*_eq_	
F	1.32492 (8)	0.12305 (7)	1.08865 (8)	0.0437 (3)	
O	0.89904 (9)	0.13207 (7)	0.84616 (8)	0.0271 (3)	
N1	0.83956 (11)	0.19499 (8)	0.67743 (9)	0.0249 (3)	
N2	0.98761 (11)	0.20522 (9)	0.61001 (10)	0.0288 (4)	
C1	0.94433 (14)	0.21182 (11)	0.68318 (12)	0.0289 (4)	
H1	0.9829 (14)	0.2262 (11)	0.7390 (12)	0.027 (5)*	
C2	0.90500 (13)	0.18153 (9)	0.54985 (11)	0.0236 (4)	
C3	0.90444 (14)	0.16519 (10)	0.46108 (12)	0.0280 (4)	
H3	0.9686 (16)	0.1703 (12)	0.4301 (13)	0.038 (6)*	
C4	0.81070 (14)	0.14160 (10)	0.41732 (12)	0.0292 (4)	
H4	0.8070 (16)	0.1292 (12)	0.3561 (15)	0.039 (6)*	
C5	0.71803 (14)	0.13502 (10)	0.45929 (12)	0.0283 (4)	
H5	0.6542 (15)	0.1181 (11)	0.4275 (13)	0.030 (5)*	
C6	0.71679 (13)	0.15155 (10)	0.54705 (12)	0.0255 (4)	
H6	0.6538 (16)	0.1488 (12)	0.5780 (13)	0.036 (5)*	
C7	0.81188 (13)	0.17443 (9)	0.59110 (11)	0.0227 (4)	
C8	0.77179 (14)	0.19141 (11)	0.74888 (12)	0.0274 (4)	
H81	0.7859 (15)	0.2378 (13)	0.7864 (13)	0.037 (6)*	
H82	0.6946 (16)	0.1908 (11)	0.7251 (13)	0.032 (5)*	
C9	0.79393 (13)	0.12350 (10)	0.80559 (11)	0.0257 (4)	
H9	0.7892 (14)	0.0767 (12)	0.7672 (13)	0.030 (5)*	
C10	0.71281 (13)	0.11680 (10)	0.87108 (11)	0.0239 (4)	
C11	0.63294 (14)	0.06463 (11)	0.85919 (12)	0.0275 (4)	
H11	0.6307 (14)	0.0317 (12)	0.8102 (13)	0.030 (5)*	
C12	0.55543 (14)	0.06074 (11)	0.91698 (12)	0.0294 (4)	
H12	0.5013 (16)	0.0250 (12)	0.9071 (13)	0.036 (5)*	
C13	0.55847 (14)	0.10849 (11)	0.98734 (12)	0.0302 (4)	
H13	0.5027 (16)	0.1064 (11)	1.0291 (13)	0.034 (5)*	
C14	0.63851 (14)	0.16059 (11)	0.99970 (12)	0.0300 (4)	
H14	0.6394 (16)	0.1942 (13)	1.0485 (14)	0.042 (6)*	
C15	0.71528 (14)	0.16487 (10)	0.94216 (12)	0.0277 (4)	
H15	0.7732 (15)	0.2003 (11)	0.9511 (12)	0.028 (5)*	
C16	0.93679 (14)	0.06654 (11)	0.89152 (13)	0.0294 (4)	
H161	0.9424 (17)	0.0256 (14)	0.8473 (15)	0.048 (6)*	
H162	0.8831 (15)	0.0495 (11)	0.9330 (13)	0.030 (5)*	
C17	1.04175 (13)	0.08265 (10)	0.94178 (11)	0.0259 (4)	
C18	1.05046 (14)	0.13885 (11)	1.00380 (12)	0.0301 (4)	
H18	0.9877 (18)	0.1682 (13)	1.0149 (14)	0.048 (6)*	
C19	1.14550 (15)	0.15275 (11)	1.05316 (13)	0.0323 (4)	
H19	1.1513 (16)	0.1897 (13)	1.0964 (14)	0.038 (6)*	
C20	1.23161 (14)	0.10960 (11)	1.03963 (12)	0.0297 (4)	
C21	1.22666 (15)	0.05391 (11)	0.97882 (12)	0.0317 (4)	
H21	1.2875 (16)	0.0263 (12)	0.9714 (13)	0.037 (6)*	
C22	1.13085 (14)	0.04091 (11)	0.92987 (12)	0.0295 (4)	
H22	1.1258 (15)	−0.0001 (12)	0.8881 (14)	0.036 (5)*	
F'	0.72117 (9)	0.07392 (7)	0.18637 (7)	0.0422 (3)	
O'	0.35374 (9)	0.09943 (7)	0.42429 (9)	0.0319 (3)	
N1'	0.32870 (11)	0.19095 (8)	0.57339 (9)	0.0250 (3)	
N2'	0.49161 (11)	0.21227 (8)	0.63993 (10)	0.0274 (3)	
C1'	0.43292 (13)	0.20470 (10)	0.56517 (12)	0.0265 (4)	
H1A	0.4581 (15)	0.2084 (11)	0.5054 (13)	0.033 (5)*	
C2'	0.42077 (13)	0.20278 (9)	0.70299 (12)	0.0242 (4)	
C3'	0.43907 (14)	0.20280 (10)	0.79409 (12)	0.0277 (4)	
H3A	0.5121 (16)	0.2104 (11)	0.8237 (13)	0.035 (5)*	
C4'	0.35328 (14)	0.19125 (11)	0.84097 (12)	0.0290 (4)	
H4A	0.3623 (15)	0.1912 (11)	0.9025 (14)	0.034 (5)*	
C5'	0.25116 (14)	0.18014 (10)	0.79938 (12)	0.0284 (4)	
H5A	0.1897 (14)	0.1719 (10)	0.8350 (12)	0.023 (5)*	
C6'	0.23161 (13)	0.17905 (10)	0.70912 (12)	0.0252 (4)	
H6A	0.1612 (15)	0.1685 (11)	0.6825 (12)	0.029 (5)*	
C7'	0.31895 (13)	0.18983 (9)	0.66247 (11)	0.0234 (4)	
C8'	0.24572 (14)	0.17291 (10)	0.50377 (12)	0.0267 (4)	
H8A	0.2466 (15)	0.2088 (12)	0.4553 (13)	0.034 (5)*	
H8B	0.1797 (16)	0.1747 (12)	0.5281 (13)	0.034 (5)*	
C9'	0.25876 (14)	0.09658 (10)	0.46651 (12)	0.0282 (4)	
H9A	0.2690 (15)	0.0595 (12)	0.5175 (13)	0.033 (5)*	
C10'	0.16396 (13)	0.07542 (10)	0.40381 (12)	0.0278 (4)	
C11'	0.10205 (14)	0.01572 (11)	0.42324 (13)	0.0311 (4)	
H11A	0.1273 (17)	−0.0142 (13)	0.4771 (15)	0.046 (6)*	
C12'	0.01201 (15)	−0.00234 (12)	0.36915 (13)	0.0356 (5)	
H12A	−0.0308 (18)	−0.0463 (14)	0.3827 (15)	0.051 (6)*	
C13'	−0.01693 (15)	0.03929 (12)	0.29548 (13)	0.0367 (5)	
H13A	−0.0804 (18)	0.0252 (13)	0.2610 (14)	0.045 (6)*	
C14'	0.04507 (16)	0.09780 (12)	0.27391 (13)	0.0380 (5)	
H14A	0.0274 (17)	0.1268 (13)	0.2198 (15)	0.046 (6)*	
C15'	0.13594 (16)	0.11604 (12)	0.32758 (13)	0.0334 (4)	
H15A	0.1795 (16)	0.1575 (13)	0.3127 (14)	0.042 (6)*	
C16'	0.38651 (15)	0.02982 (11)	0.39365 (14)	0.0313 (4)	
H16A	0.3236 (17)	0.0054 (12)	0.3565 (14)	0.041 (6)*	
H16B	0.4113 (16)	−0.0028 (13)	0.4487 (14)	0.041 (6)*	
C17'	0.47598 (13)	0.04194 (10)	0.33826 (11)	0.0263 (4)	
C18'	0.47972 (15)	0.10525 (11)	0.28687 (12)	0.0295 (4)	
H18A	0.4234 (15)	0.1408 (12)	0.2871 (13)	0.031 (5)*	
C19'	0.56238 (15)	0.11670 (11)	0.23553 (12)	0.0316 (4)	
H19A	0.5682 (16)	0.1634 (13)	0.2001 (14)	0.040 (6)*	
C20'	0.63892 (14)	0.06312 (11)	0.23568 (12)	0.0295 (4)	
C21'	0.63728 (15)	−0.00091 (11)	0.28360 (13)	0.0330 (4)	
H21A	0.6947 (18)	−0.0365 (14)	0.2804 (14)	0.049 (6)*	
C22'	0.55530 (14)	−0.01061 (11)	0.33574 (12)	0.0300 (4)	
H22A	0.5502 (16)	−0.0561 (13)	0.3701 (14)	0.039 (6)*	

### Atomic displacement parameters (Å^2^)

**Table:**

	*U*^11^	*U*^22^	*U*^33^	*U*^12^	*U*^13^	*U*^23^
F	0.0279 (6)	0.0494 (8)	0.0519 (7)	−0.0033 (5)	−0.0053 (5)	0.0001 (6)
O	0.0248 (6)	0.0294 (7)	0.0266 (6)	0.0005 (5)	0.0007 (5)	0.0048 (5)
N1	0.0223 (7)	0.0297 (8)	0.0225 (7)	−0.0015 (6)	0.0014 (6)	0.0024 (6)
N2	0.0240 (7)	0.0334 (9)	0.0287 (8)	−0.0050 (6)	0.0007 (6)	0.0060 (7)
C1	0.0260 (9)	0.0331 (11)	0.0269 (10)	−0.0049 (7)	−0.0012 (8)	0.0041 (8)
C2	0.0214 (8)	0.0222 (9)	0.0272 (9)	−0.0001 (7)	0.0019 (7)	0.0047 (7)
C3	0.0272 (9)	0.0295 (10)	0.0281 (10)	0.0026 (8)	0.0073 (7)	0.0052 (8)
C4	0.0342 (10)	0.0290 (10)	0.0244 (9)	0.0038 (8)	0.0024 (7)	−0.0012 (8)
C5	0.0266 (9)	0.0277 (10)	0.0295 (10)	−0.0008 (7)	−0.0022 (7)	−0.0001 (8)
C6	0.0223 (8)	0.0254 (9)	0.0286 (9)	−0.0003 (7)	0.0025 (7)	0.0022 (7)
C7	0.0248 (8)	0.0220 (9)	0.0212 (8)	0.0021 (7)	0.0017 (7)	0.0027 (7)
C8	0.0262 (9)	0.0321 (10)	0.0241 (9)	0.0010 (8)	0.0045 (7)	0.0021 (8)
C9	0.0237 (9)	0.0295 (10)	0.0235 (9)	0.0007 (7)	0.0013 (7)	0.0003 (8)
C10	0.0223 (8)	0.0270 (9)	0.0221 (9)	0.0022 (7)	0.0010 (6)	0.0026 (7)
C11	0.0292 (9)	0.0285 (10)	0.0242 (9)	−0.0014 (7)	−0.0004 (7)	0.0001 (8)
C12	0.0238 (9)	0.0318 (10)	0.0318 (10)	−0.0051 (8)	−0.0010 (7)	0.0077 (8)
C13	0.0271 (9)	0.0342 (11)	0.0297 (10)	0.0036 (8)	0.0054 (8)	0.0082 (8)
C14	0.0323 (10)	0.0306 (10)	0.0279 (10)	0.0013 (8)	0.0069 (8)	0.0010 (8)
C15	0.0285 (9)	0.0269 (10)	0.0277 (9)	−0.0036 (8)	0.0026 (7)	0.0003 (8)
C16	0.0293 (9)	0.0268 (10)	0.0315 (10)	0.0040 (7)	0.0006 (8)	0.0030 (8)
C17	0.0269 (9)	0.0259 (9)	0.0252 (9)	0.0017 (7)	0.0045 (7)	0.0053 (7)
C18	0.0280 (9)	0.0291 (10)	0.0340 (10)	0.0032 (8)	0.0065 (8)	−0.0001 (8)
C19	0.0344 (10)	0.0299 (11)	0.0326 (10)	−0.0023 (8)	0.0029 (8)	−0.0018 (9)
C20	0.0236 (9)	0.0330 (10)	0.0319 (10)	−0.0035 (8)	−0.0003 (7)	0.0071 (8)
C21	0.0285 (9)	0.0352 (11)	0.0322 (10)	0.0083 (8)	0.0072 (8)	0.0070 (8)
C22	0.0343 (10)	0.0318 (10)	0.0224 (9)	0.0069 (8)	0.0039 (7)	0.0034 (8)
F'	0.0391 (6)	0.0524 (8)	0.0372 (6)	0.0111 (5)	0.0142 (5)	0.0048 (6)
O'	0.0283 (7)	0.0285 (7)	0.0400 (8)	−0.0007 (5)	0.0087 (6)	−0.0077 (6)
N1'	0.0237 (7)	0.0277 (8)	0.0237 (8)	−0.0014 (6)	0.0032 (6)	−0.0005 (6)
N2'	0.0243 (7)	0.0276 (8)	0.0307 (8)	−0.0024 (6)	0.0043 (6)	−0.0004 (6)
C1'	0.0255 (9)	0.0256 (9)	0.0289 (10)	−0.0011 (7)	0.0056 (7)	−0.0002 (7)
C2'	0.0235 (8)	0.0197 (9)	0.0296 (9)	0.0003 (7)	0.0034 (7)	−0.0005 (7)
C3'	0.0257 (9)	0.0253 (10)	0.0310 (10)	0.0012 (7)	−0.0018 (7)	−0.0015 (8)
C4'	0.0329 (10)	0.0288 (10)	0.0251 (10)	0.0055 (8)	0.0020 (8)	0.0003 (8)
C5'	0.0282 (9)	0.0269 (10)	0.0310 (10)	0.0029 (7)	0.0077 (8)	0.0018 (8)
C6'	0.0216 (8)	0.0241 (9)	0.0298 (10)	0.0009 (7)	0.0030 (7)	−0.0016 (7)
C7'	0.0250 (8)	0.0191 (8)	0.0261 (9)	0.0026 (7)	0.0020 (7)	−0.0005 (7)
C8'	0.0249 (9)	0.0305 (10)	0.0244 (9)	−0.0005 (7)	0.0007 (7)	−0.0009 (8)
C9'	0.0256 (9)	0.0286 (10)	0.0308 (10)	−0.0014 (7)	0.0041 (7)	0.0003 (8)
C10'	0.0265 (9)	0.0299 (10)	0.0269 (9)	0.0031 (7)	0.0023 (7)	−0.0064 (8)
C11'	0.0317 (10)	0.0282 (10)	0.0333 (10)	0.0015 (8)	0.0025 (8)	−0.0043 (8)
C12'	0.0332 (10)	0.0344 (11)	0.0399 (11)	−0.0034 (9)	0.0060 (8)	−0.0106 (9)
C13'	0.0291 (10)	0.0464 (13)	0.0337 (11)	0.0032 (9)	−0.0011 (8)	−0.0144 (9)
C14'	0.0444 (12)	0.0448 (13)	0.0241 (10)	0.0092 (10)	0.0001 (8)	−0.0034 (9)
C15'	0.0384 (11)	0.0324 (11)	0.0302 (10)	−0.0002 (9)	0.0074 (8)	−0.0035 (8)
C16'	0.0297 (9)	0.0264 (10)	0.0381 (11)	0.0013 (8)	0.0049 (8)	−0.0026 (8)
C17'	0.0263 (9)	0.0253 (9)	0.0265 (9)	0.0006 (7)	−0.0011 (7)	−0.0049 (7)
C18'	0.0295 (9)	0.0300 (10)	0.0286 (10)	0.0074 (8)	0.0011 (7)	−0.0026 (8)
C19'	0.0365 (10)	0.0316 (11)	0.0265 (10)	0.0066 (8)	0.0032 (8)	0.0013 (8)
C20'	0.0283 (9)	0.0362 (11)	0.0245 (9)	0.0031 (8)	0.0048 (7)	−0.0029 (8)
C21'	0.0323 (10)	0.0306 (11)	0.0359 (11)	0.0080 (8)	0.0028 (8)	−0.0049 (8)
C22'	0.0336 (10)	0.0237 (10)	0.0323 (10)	0.0027 (8)	0.0006 (8)	−0.0013 (8)

### Geometric parameters (Å, °)

**Table:**

F—C20	1.360 (2)	F'—C20'	1.364 (2)
O—C9	1.423 (2)	O'—C9'	1.426 (2)
O—C16	1.435 (2)	O'—C16'	1.423 (2)
N1—C1	1.359 (2)	N1'—C1'	1.365 (2)
N1—C7	1.388 (2)	N1'—C7'	1.386 (2)
N1—C8	1.462 (2)	N1'—C8'	1.461 (2)
N2—C1	1.305 (2)	N2'—C1'	1.312 (2)
N2—C2	1.394 (2)	N2'—C2'	1.397 (2)
C1—H1	0.980 (19)	C1'—H1A	1.00 (2)
C2—C3	1.394 (3)	C2'—C7'	1.397 (2)
C2—C7	1.402 (2)	C3'—C2'	1.395 (3)
C3—H3	0.99 (2)	C3'—C4'	1.380 (3)
C4—C3	1.375 (3)	C3'—H3A	1.00 (2)
C4—C5	1.402 (3)	C4'—C5'	1.401 (3)
C4—H4	0.96 (2)	C4'—H4A	0.94 (2)
C5—H5	0.95 (2)	C5'—C6'	1.383 (3)
C6—C5	1.382 (3)	C5'—H5A	1.008 (18)
C6—H6	0.97 (2)	C6'—H6A	0.964 (19)
C7—C6	1.387 (2)	C7'—C6'	1.393 (2)
C8—H81	1.03 (2)	C8'—C9'	1.513 (3)
C8—H82	1.011 (19)	C8'—H8A	0.99 (2)
C9—C8	1.518 (3)	C8'—H8B	0.95 (2)
C9—C10	1.514 (2)	C9'—C10'	1.513 (2)
C9—H9	1.03 (2)	C9'—H9A	1.03 (2)
C10—C15	1.395 (3)	C10'—C11'	1.387 (3)
C11—C10	1.385 (3)	C10'—C15'	1.398 (3)
C11—C12	1.391 (3)	C11'—C12'	1.383 (3)
C11—H11	0.96 (2)	C11'—H11A	1.01 (2)
C12—C13	1.382 (3)	C12'—H12A	1.00 (2)
C12—H12	0.95 (2)	C13'—C12'	1.379 (3)
C13—H13	1.00 (2)	C13'—C14'	1.381 (3)
C14—C13	1.386 (3)	C13'—H13A	0.95 (2)
C14—C15	1.382 (3)	C14'—H14A	0.99 (2)
C14—H14	0.97 (2)	C15'—C14'	1.389 (3)
C15—H15	0.97 (2)	C15'—H15A	0.97 (2)
C16—H161	1.01 (2)	C16'—H16A	1.03 (2)
C16—H162	1.03 (2)	C16'—H16B	1.05 (2)
C17—C16	1.500 (2)	C17'—C16'	1.502 (3)
C17—C18	1.391 (3)	C17'—C18'	1.396 (3)
C17—C22	1.388 (2)	C17'—C22'	1.389 (3)
C18—C19	1.382 (3)	C18'—C19'	1.390 (3)
C18—H18	0.99 (2)	C18'—H18A	0.96 (2)
C19—H19	0.94 (2)	C19'—H19A	1.01 (2)
C20—C19	1.377 (3)	C20'—C19'	1.374 (3)
C20—C21	1.372 (3)	C20'—C21'	1.376 (3)
C21—C22	1.384 (3)	C21'—C22'	1.386 (3)
C21—H21	0.94 (2)	C21'—H21A	0.98 (2)
C22—H22	0.98 (2)	C22'—H22A	0.98 (2)
			
C9—O—C16	112.02 (13)	C16'—O'—C9'	113.99 (14)
C1—N1—C7	105.96 (14)	C1'—N1'—C7'	106.09 (14)
C1—N1—C8	127.37 (15)	C1'—N1'—C8'	127.44 (15)
C7—N1—C8	126.37 (14)	C7'—N1'—C8'	126.15 (14)
C1—N2—C2	104.19 (14)	C1'—N2'—C2'	104.19 (14)
N1—C1—H1	121.1 (11)	N1'—C1'—H1A	119.8 (11)
N2—C1—N1	114.66 (16)	N2'—C1'—N1'	114.16 (16)
N2—C1—H1	124.2 (11)	N2'—C1'—H1A	126.0 (11)
N2—C2—C7	109.96 (15)	N2'—C2'—C7'	110.07 (15)
C3—C2—N2	130.02 (16)	C3'—C2'—N2'	129.88 (16)
C3—C2—C7	120.02 (15)	C3'—C2'—C7'	120.03 (16)
C2—C3—H3	121.8 (12)	C2'—C3'—H3A	120.6 (11)
C4—C3—C2	117.60 (16)	C4'—C3'—C2'	117.52 (16)
C4—C3—H3	120.6 (12)	C4'—C3'—H3A	121.8 (11)
C3—C4—C5	121.79 (17)	C3'—C4'—C5'	121.77 (17)
C3—C4—H4	120.3 (12)	C3'—C4'—H4A	120.1 (12)
C5—C4—H4	117.9 (12)	C5'—C4'—H4A	118.2 (12)
C4—C5—H5	120.1 (12)	C4'—C5'—H5A	120.3 (10)
C6—C5—C4	121.45 (16)	C6'—C5'—C4'	121.58 (17)
C6—C5—H5	118.5 (12)	C6'—C5'—H5A	118.1 (10)
C5—C6—C7	116.49 (16)	C5'—C6'—C7'	116.17 (16)
C5—C6—H6	123.8 (12)	C5'—C6'—H6A	119.6 (11)
C7—C6—H6	119.7 (12)	C7'—C6'—H6A	124.2 (11)
N1—C7—C2	105.22 (14)	N1'—C7'—C6'	131.61 (15)
C6—C7—N1	132.15 (16)	N1'—C7'—C2'	105.49 (14)
C6—C7—C2	122.63 (16)	C6'—C7'—C2'	122.90 (16)
N1—C8—C9	112.17 (15)	N1'—C8'—C9'	112.54 (15)
N1—C8—H81	107.6 (11)	N1'—C8'—H8A	110.0 (11)
N1—C8—H82	110.6 (11)	N1'—C8'—H8B	107.7 (12)
C9—C8—H81	109.3 (11)	C9'—C8'—H8A	107.9 (12)
C9—C8—H82	108.4 (12)	C9'—C8'—H8B	108.2 (13)
H81—C8—H82	108.7 (16)	H8A—C8'—H8B	110.5 (17)
O—C9—C8	105.91 (14)	O'—C9'—C8'	105.57 (14)
O—C9—C10	112.84 (14)	O'—C9'—C10'	112.01 (15)
O—C9—H9	109.7 (10)	O'—C9'—H9A	108.7 (11)
C8—C9—H9	110.0 (11)	C8'—C9'—C10'	111.24 (15)
C10—C9—C8	110.29 (14)	C8'—C9'—H9A	108.7 (11)
C10—C9—H9	108.0 (11)	C10'—C9'—H9A	110.4 (11)
C11—C10—C9	120.31 (16)	C11'—C10'—C15'	119.25 (17)
C11—C10—C15	119.24 (16)	C11'—C10'—C9'	119.50 (17)
C15—C10—C9	120.40 (16)	C15'—C10'—C9'	121.22 (17)
C10—C11—C12	120.38 (17)	C10'—C11'—H11A	117.0 (13)
C10—C11—H11	119.5 (12)	C12'—C11'—C10'	120.49 (19)
C12—C11—H11	120.1 (12)	C12'—C11'—H11A	122.5 (13)
C11—C12—H12	118.8 (13)	C11'—C12'—H12A	119.9 (13)
C13—C12—C11	120.05 (17)	C13'—C12'—C11'	120.0 (2)
C13—C12—H12	121.2 (13)	C13'—C12'—H12A	120.1 (13)
C12—C13—C14	119.77 (17)	C12'—C13'—C14'	120.38 (18)
C12—C13—H13	120.6 (12)	C12'—C13'—H13A	116.8 (14)
C14—C13—H13	119.6 (12)	C14'—C13'—H13A	122.8 (14)
C13—C14—H14	119.2 (13)	C13'—C14'—C15'	120.01 (19)
C15—C14—C13	120.33 (18)	C13'—C14'—H14A	121.5 (13)
C15—C14—H14	120.5 (13)	C15'—C14'—H14A	118.5 (13)
C10—C15—H15	118.6 (11)	C10'—C15'—H15A	120.0 (13)
C14—C15—C10	120.22 (17)	C14'—C15'—C10'	119.85 (19)
C14—C15—H15	121.2 (11)	C14'—C15'—H15A	120.1 (12)
O—C16—C17	108.96 (15)	O'—C16'—C17'	108.67 (15)
O—C16—H161	108.9 (13)	O'—C16'—H16A	109.1 (12)
O—C16—H162	109.9 (11)	O'—C16'—H16B	107.8 (12)
C17—C16—H162	110.5 (10)	C17'—C16'—H16A	109.7 (12)
C17—C16—H161	111.6 (12)	C17'—C16'—H16B	110.8 (11)
H162—C16—H161	106.9 (17)	H16A—C16'—H16B	110.7 (17)
C18—C17—C16	120.02 (16)	C18'—C17'—C16'	120.72 (16)
C22—C17—C18	118.64 (17)	C22'—C17'—C18'	118.82 (17)
C22—C17—C16	121.30 (17)	C22'—C17'—C16'	120.45 (17)
C17—C18—H18	120.2 (13)	C17'—C18'—H18A	118.8 (12)
C19—C18—C17	120.93 (17)	C19'—C18'—C17'	120.91 (17)
C19—C18—H18	118.8 (13)	C19'—C18'—H18A	120.3 (12)
C18—C19—H19	121.3 (13)	C18'—C19'—H19A	121.9 (12)
C20—C19—C18	118.45 (19)	C20'—C19'—C18'	117.87 (18)
C20—C19—H19	120.2 (13)	C20'—C19'—H19A	120.2 (12)
F—C20—C19	118.38 (17)	F'—C20'—C19'	118.57 (17)
F—C20—C21	119.09 (16)	F'—C20'—C21'	118.13 (16)
C21—C20—C19	122.53 (17)	C19'—C20'—C21'	123.30 (18)
C20—C21—C22	118.19 (17)	C20'—C21'—C22'	117.92 (18)
C20—C21—H21	119.6 (13)	C20'—C21'—H21A	118.6 (14)
C22—C21—H21	122.2 (13)	C22'—C21'—H21A	123.5 (14)
C17—C22—H22	119.9 (12)	C17'—C22'—H22A	118.2 (12)
C21—C22—C17	121.26 (18)	C21'—C22'—C17'	121.14 (18)
C21—C22—H22	118.8 (12)	C21'—C22'—H22A	120.6 (12)
			
C16—O—C9—C8	−171.09 (14)	C16'—O'—C9'—C8'	172.99 (15)
C16—O—C9—C10	68.15 (19)	C16'—O'—C9'—C10'	−65.8 (2)
C9—O—C16—C17	−172.87 (14)	C9'—O'—C16'—C17'	170.86 (14)
C7—N1—C1—N2	0.7 (2)	C7'—N1'—C1'—N2'	0.3 (2)
C8—N1—C1—N2	174.73 (17)	C8'—N1'—C1'—N2'	−173.60 (17)
C1—N1—C7—C2	−0.70 (18)	C1'—N1'—C7'—C6'	179.34 (18)
C1—N1—C7—C6	179.78 (19)	C8'—N1'—C7'—C6'	−6.7 (3)
C8—N1—C7—C2	−174.84 (16)	C1'—N1'—C7'—C2'	−0.44 (18)
C8—N1—C7—C6	5.6 (3)	C8'—N1'—C7'—C2'	173.54 (16)
C1—N1—C8—C9	−73.6 (2)	C1'—N1'—C8'—C9'	70.5 (2)
C7—N1—C8—C9	99.33 (19)	C7'—N1'—C8'—C9'	−102.2 (2)
C2—N2—C1—N1	−0.3 (2)	C2'—N2'—C1'—N1'	0.0 (2)
C1—N2—C2—C3	−179.88 (19)	C1'—N2'—C2'—C3'	177.92 (19)
C1—N2—C2—C7	−0.15 (19)	C1'—N2'—C2'—C7'	−0.30 (19)
N2—C2—C3—C4	179.03 (18)	C3'—C2'—C7'—N1'	−177.96 (16)
C7—C2—C3—C4	−0.7 (3)	N2'—C2'—C7'—N1'	0.47 (19)
N2—C2—C7—N1	0.54 (19)	C3'—C2'—C7'—C6'	2.2 (3)
N2—C2—C7—C6	−179.88 (16)	N2'—C2'—C7'—C6'	−179.33 (16)
C3—C2—C7—N1	−179.70 (16)	C4'—C3'—C2'—N2'	−179.40 (17)
C3—C2—C7—C6	−0.1 (3)	C4'—C3'—C2'—C7'	−1.3 (3)
C5—C4—C3—C2	0.9 (3)	C2'—C3'—C4'—C5'	−0.2 (3)
C3—C4—C5—C6	−0.4 (3)	C3'—C4'—C5'—C6'	1.0 (3)
C7—C6—C5—C4	−0.4 (3)	C4'—C5'—C6'—C7'	−0.2 (3)
N1—C7—C6—C5	−179.90 (18)	N1'—C7'—C6'—C5'	178.82 (18)
C2—C7—C6—C5	0.6 (3)	C2'—C7'—C6'—C5'	−1.4 (3)
O—C9—C8—N1	65.00 (18)	N1'—C8'—C9'—O'	−66.22 (19)
C10—C9—C8—N1	−172.60 (14)	N1'—C8'—C9'—C10'	172.07 (15)
O—C9—C10—C11	−136.59 (17)	O'—C9'—C10'—C11'	125.14 (18)
O—C9—C10—C15	45.8 (2)	C8'—C9'—C10'—C11'	−116.98 (19)
C8—C9—C10—C11	105.18 (19)	O'—C9'—C10'—C15'	−56.5 (2)
C8—C9—C10—C15	−72.4 (2)	C8'—C9'—C10'—C15'	61.4 (2)
C9—C10—C15—C14	177.27 (16)	C9'—C10'—C11'—C12'	176.72 (17)
C11—C10—C15—C14	−0.3 (3)	C15'—C10'—C11'—C12'	−1.7 (3)
C12—C11—C10—C9	−176.91 (16)	C9'—C10'—C15'—C14'	−176.33 (17)
C12—C11—C10—C15	0.7 (3)	C11'—C10'—C15'—C14'	2.1 (3)
C10—C11—C12—C13	−0.8 (3)	C10'—C11'—C12'—C13'	−0.2 (3)
C11—C12—C13—C14	0.5 (3)	C14'—C13'—C12'—C11'	1.8 (3)
C15—C14—C13—C12	−0.1 (3)	C12'—C13'—C14'—C15'	−1.4 (3)
C13—C14—C15—C10	0.1 (3)	C10'—C15'—C14'—C13'	−0.5 (3)
C18—C17—C16—O	57.8 (2)	C18'—C17'—C16'—O'	−33.7 (2)
C22—C17—C16—O	−124.31 (18)	C22'—C17'—C16'—O'	147.83 (17)
C16—C17—C18—C19	177.53 (17)	C16'—C17'—C18'—C19'	179.98 (17)
C22—C17—C18—C19	−0.4 (3)	C22'—C17'—C18'—C19'	−1.5 (3)
C16—C17—C22—C21	−177.36 (17)	C18'—C17'—C22'—C21'	0.2 (3)
C18—C17—C22—C21	0.6 (3)	C16'—C17'—C22'—C21'	178.70 (17)
C17—C18—C19—C20	−0.1 (3)	C17'—C18'—C19'—C20'	1.3 (3)
F—C20—C19—C18	−179.59 (16)	F'—C20'—C19'—C18'	−179.30 (16)
C21—C20—C19—C18	0.4 (3)	C21'—C20'—C19'—C18'	0.3 (3)
F—C20—C21—C22	179.72 (16)	F'—C20'—C21'—C22'	178.03 (16)
C19—C20—C21—C22	−0.3 (3)	C19'—C20'—C21'—C22'	−1.6 (3)
C20—C21—C22—C17	−0.2 (3)	C20'—C21'—C22'—C17'	1.3 (3)

### Hydrogen-bond geometry (Å, °)

**Table:**

*D*—H···*A*	*D*—H	H···*A*	*D*···*A*	*D*—H···*A*
C6—H6···N2'	0.97 (2)	2.62 (2)	3.498 (2)	150.5 (17)
C6'—H6A···N2^i^	0.964 (19)	2.459 (19)	3.345 (2)	152.7 (16)
C13—H13···F^i^	1.00 (2)	2.54 (2)	3.493 (2)	159.6 (16)
C19'—H19A···N2'^ii^	1.01 (2)	2.59 (2)	3.509 (2)	151.1 (16)
C5—H5···Cg8^iii^	0.95 (2)	2.662 (19)	3.514 (2)	149.0 (15)
C22'—H22A···Cg2^iv^	0.98 (2)	2.62 (9)	3.518 (2)	141.9 (16)

Symmetry codes: (i) *x*−1, *y*, *z*; (ii) *x*, −*y*+1/2, *z*−1/2; (iii) *x*, −*y*−1/2, *z*−3/2; (iv) −*x*+1, −*y*, −*z*+1.

## References

[bb1] Brammer, K. W. & Feczko, J. M. (1988). In *Antifungal Drugs*, edited by V. St. Georgiev, pp. 561–563. New York Academy of Science.

[bb2] Caira, M. R., Alkhamis, K. A. & Obaidat, R. M. (2004). *J. Pharm. Sci.* **93**, 601–611. 10.1002/jps.10541 14762899

[bb3] Farrugia, L. J. (1997). *J. Appl. Cryst.* **30**, 565.

[bb4] Farrugia, L. J. (1999). *J. Appl. Cryst.* **32**, 837–838.

[bb5] Freer, A. A., Pearson, A. & Salole, E. G. (1986). *Acta Cryst.* C**42**, 1350–1352.

[bb6] Hooft, R. W. W. (1998). *COLLECT* Nonius BV, Delft, The Netherlands.

[bb7] Otwinowski, Z. & Minor, W. (1997). *Methods in Enzimology*, Vol. 276, *Macromolecular Crystallography*, Part A, edited by C. W. Carter Jr & R. M. Sweet, pp. 307–326. New York: Academic Press.

[bb8] Özel Güven, Ö., Erdoğan, T., Göker, H. & Yıldız, S. (2007*a*). *Bioorg. Med. Chem. Lett.* **17**, 2233–2236. 10.1016/j.bmcl.2007.01.061 17289382

[bb9] Özel Güven, Ö., Erdoğan, T., Göker, H. & Yıldız, S. (2007*b*). *J. Heterocycl. Chem.* **44**, 731–734.

[bb10] Peeters, O. M., Blaton, N. M. & De Ranter, C. J. (1979*a*). *Acta Cryst.* B**35**, 2461–2464.

[bb11] Peeters, O. M., Blaton, N. M. & De Ranter, C. J. (1979*b*). *Bull. Soc. Chim. Belg.* **88**, 265–272.

[bb12] Peeters, O. M., Blaton, N. M. & De Ranter, C. J. (1996). *Acta Cryst.* C**52**, 2225–2229.

[bb13] Sheldrick, G. M. (2007). *SADABS* Bruker AXS Inc., Madison, Wisconsin, USA.

[bb14] Sheldrick, G. M. (2008). *Acta Cryst.* A**64**, 112–122. 10.1107/S0108767307043930 18156677

[bb15] Song, H. & Shin, H.-S. (1998). *Acta Cryst.* C**54**, 1675–1677. 10.1107/s0108270198006386 9857476

[bb16] Spek, A. L. (2003). *J. Appl. Cryst.* **36**, 7–13.

